# RNA Vaccination Therapy: Advances in an Emerging Field

**DOI:** 10.1155/2016/9703914

**Published:** 2016-02-25

**Authors:** Sebastian Kreiter, Mustafa Diken, Steve Pascolo, Smita K. Nair, Kris M. Thielemans, Andrew Geall

**Affiliations:** ^1^TRON-Translational Oncology at the University Medical Center of the Johannes Gutenberg University gGmbH, 55131 Mainz, Germany; ^2^Department of Dermatology, University Hospital of Zurich, 8091 Zurich, Switzerland; ^3^Department of Surgery, Duke University Medical Center, Durham, NC 27710, USA; ^4^Department of Pathology, Duke University Medical Center, Durham, NC 27710, USA; ^5^Laboratory of Molecular and Cellular Therapy (LMCT), Vrije Universiteit Brussel (VUB), 1090 Brussels, Belgium; ^6^Avidity NanoMedicines, La Jolla, CA 92037, USA

After more than two decades of research, the efforts to translate the concept of RNA based vaccination have reached a critical mass. Several preclinical and clinical projects located in the academic or industrial setting are underway and the coming years will allow us to get broad insight into clinical feasibility, safety, and first efficacy data. It can be anticipated that some RNA based vaccines will be approved within the near future.

The use of* in vitro* transcribed RNA is now viewed as an attractive approach for vaccination therapies, with several features contributing to its favorable characteristics. RNA allows expression of molecularly well-defined proteins and its half-life can be steered through modifications in the RNA backbone. Moreover, unlike DNA, RNA does not need to enter the nucleus during transfection and there is no risk of integration into the genome, assuring safety through transient activity. Rapid design and synthesis in response to demand, accompanied by inexpensive pharmaceutical production, are additional features facilitating its clinical translation.

The seminal work of Wolff et al. which showed that RNA injected directly into skeletal muscle can lead to protein expression opened the era of RNA based therapeutics [1]. This observation was followed by Martinon et al. and Conry et al. who performed the first vaccinations with viral- and cancer-antigen encoding RNA, respectively, and elicited antigen-specific immune responses [2, 3]. RNA based vaccination was also carried out by* ex vivo* transfection of mRNA into autologous dendritic cells (DCs) which was initially described by Boczkowski et al. [4]. Along with the introduction of highly efficient transfection methods for RNA [5], several preclinical and clinical studies showed the safety and efficacy of this RNA based vaccination strategy [6]. In a different setting, Hoerr et al. proved that direct injection of naked or protamine-protected RNA intradermally can lead to induction of T cell and antibody responses in preclinical models and then translated the approach into a clinical setting [7–10]. Personalized cancer vaccination with RNA and intravenous delivery of liposome-complexed RNA [11, 12] are other recent promising strategies that have reached the clinical stage. In addition to cancer, other disease settings such as infectious diseases as well as allergy were also shown to benefit from RNA based vaccination [13–15].

In this special issue, a number of papers will illustrate and summarize the advances in this emerging field. M. A. McNamara et al. will provide a comprehensive review on RNA based vaccines in cancer immunotherapy, which is further detailed for the use of mutanome engineered RNA by M. Vormehr et al. These will be complemented by a review from K. K. L. Phua describing targeted delivery systems for RNA based nanoparticle tumor vaccines. Other contributions will describe RNA based methods for* in vitro* analytics such as cytotoxicity (T. A. Omokoko et al.) or effects of RNA on transcriptome of DCs (S. Hoyer et al.). Finally, E. Hattinger et al. will also demonstrate, with a different disease focus, the efficacy of prophylactic RNA vaccination against allergy.

In conclusion, this special issue covers many aspects of RNA based vaccines. As RNA based vaccination is not the only application of the RNA technology (RNA based protein replacement, immunomodulation, and cellular therapy are further promising fields of development), we hope to have sparked the readers interest in RNA based therapies in general.

*Sebastian Kreiter* *Sebastian Kreiter* *Mustafa Diken* *Mustafa Diken* *Steve Pascolo* *Steve Pascolo* *Smita K. Nair* *Smita K. Nair* *Kris M. Thielemans* *Kris M. Thielemans* *Andrew Geall* *Andrew Geall*
